# Molecular profiling of immunoglobulin heavy-chain gene rearrangements unveils new potential prognostic markers for multiple myeloma patients

**DOI:** 10.1038/s41408-020-0283-8

**Published:** 2020-02-06

**Authors:** Alejandro Medina, Cristina Jiménez, M. Eugenia Sarasquete, Marcos González, M. Carmen Chillón, Ana Balanzategui, Isabel Prieto-Conde, María García-Álvarez, Noemí Puig, Verónica González-Calle, Miguel Alcoceba, Isabel Cuenca, Santiago Barrio, Fernando Escalante, Norma C. Gutiérrez, Mercedes Gironella, Miguel T. Hernández, Anna Sureda, Albert Oriol, Joan Bladé, Juan-José Lahuerta, Jesús F. San Miguel, María-Victoria Mateos, Joaquín Martínez-López, María-José Calasanz, Ramón García-Sanz

**Affiliations:** 1 Hospital Universitario de Salamanca (HUSAL), IBSAL, IBMCC (USAL-CSIC), CIBERONC, Salamanca, Spain; 2 Hospital 12 de Octubre, CIBERONC, Madrid, Spain; 3 Complejo Hospitalario, León, Spain; 4 Hospital Vall d’Hebrón, Barcelona, Spain; 5 Hospital Universitario de Canarias, La Laguna, Spain; 6 Hospital Duran i Reynals, Institut Català d’Oncología (ICO), L’Hospitalet de Llobregat, Barcelona, Spain; 7 Hospital Germans Trias i Pujol, Institut Català d’Oncología (ICO), Institut Josep Carreras, Badalona, Spain; 8 Hospital Clínic i Provincial, Institut de Investicacions Biomediques August Pi i Sunyer (IDIBAPS), Barcelona, Spain; 9 Clínica Universidad de Navarra (CUN), Centro de Investigación Médica Aplicada, IDISNA, CIBERONC, Pamplona, Spain

**Keywords:** Genetics research, Myeloma, Genetics research, Myeloma

## Abstract

Multiple myeloma is a heterogeneous disease whose pathogenesis has not been completely elucidated. Although B-cell receptors play a crucial role in myeloma pathogenesis, the impact of clonal immunoglobulin heavy-chain features in the outcome has not been extensively explored. Here we present the characterization of complete heavy-chain gene rearrangements in 413 myeloma patients treated in Spanish trials, including 113 patients characterized by next-generation sequencing. Compared to the normal B-cell repertoire, gene selection was biased in myeloma, with significant overrepresentation of *IGHV3*, *IGHD2* and *IGHD3*, as well as *IGHJ4* gene groups. Hypermutation was high in our patients (median: 8.8%). Interestingly, regarding patients who are not candidates for transplantation, a high hypermutation rate (≥7%) and the use of *IGHD2* and *IGHD3* groups were associated with improved prognostic features and longer survival rates in the univariate analyses. Multivariate analysis revealed prolonged progression-free survival rates for patients using *IGHD2/IGHD3* groups (HR: 0.552, 95% CI: 0.361−0.845, *p* = 0.006), as well as prolonged overall survival rates for patients with hypermutation ≥7% (HR: 0.291, 95% CI: 0.137−0.618, *p* = 0.001). Our results provide new insights into the molecular characterization of multiple myeloma, highlighting the need to evaluate some of these clonal rearrangement characteristics as new potential prognostic markers.

## Introduction

B-cell lymphoproliferative disorders are caused by the expansion of a pathological clone at a specific stage of differentiation reflected in the B-cell receptor (BCR). Through multiple, hierarchically structured events, BCR genes from each B cell are assembled in a particular shape that confers an extremely high diversity to the B-cell repertoire to recognize different antigens^1^. *IGHV*, *IGHD* and *IGHJ* immunoglobulin genes go through recombination during early B-cell ontogeny that takes place in the bone marrow (BM), an entirely random, antigen-independent process driven by the Recombination-Activating Genes (*RAG1* and *RAG2*)^2^ and Terminal deoxynucleotidyl Transferase (*TdT*)^3^. In humans, there are more than 50 *IGHV* genes, 27 *IGHD* genes and 6 *IGHJ* genes. *IGHV* and *IGHD* genes are clustered into seven different groups^4^. During B-cell differentiation, one allele is rearranged to produce a complete heavy-chain rearrangement (IGH) with or without the rearrangement of the second *IGH* allele^5^. This means that one or two *IGH* rearrangements can be found in each individual B cell, although only one of them is expressed as a functional protein^6^. Immunoglobulin-expressing B cells then migrate to germinal centers to undergo two antigen-dependent processes: somatic hypermutation (SHM) and class-switch recombination (CSR)^7^.

As a clonal B-cell malignancy, multiple myeloma (MM) arises from a single cell, that has been recognized as a post-germinal center, IgA/IgG switched B cell in several studies^8^. *VDJH* usage, Complementarity-determining Region 3 (CDR3) composition and somatic hypermutation (SHM) levels have been studied for the vast majority of B-cell malignancies, as well as for normal, healthy B cells^9–12^. The most interesting finding to date is the close association between SHM rate and the outcome in chronic lymphocytic leukemia (CLL), since a high SHM rate (>2%) is associated with good prognosis^13^. Another interesting observation is the presence of stereotyped immunoglobulin receptors, not only in CLL^14^ but also in mantle-cell lymphoma (MCL) or marginal zone lymphoma (MZL), which led researchers to infer that this was a common phenomenon for all mature B-cell tumors with potential prognostic and therapeutic implications^15^. Several studies performed with MM patients treated with conventional chemotherapy did not find any correlation between prognosis and *VDJH* gene usage, CDR3 amino acid composition or SHM rates^16–18^. However, most series often included an insufficient number of heterogeneously treated patients, which makes any conclusion difficult to be inferred.

Here, we present the largest-to-date analysis of the *VDJH* repertoire in multiple myeloma, consisting in biological and clinical data from 413 patients diagnosed and treated according to the Spanish Myeloma Group (GEM-PETHEMA) protocols. Data were used for a comprehensive investigation of *VDJH* rearrangement characteristics, including *IGHV*, *IGHD* and *IGHJ* gene usage, SHM level and distribution, or complementarity-determining region (CDRs) and framework region (FWRs) length and composition. We also tried to identify the presence of potential clusters of stereotyped receptors, assessing potential relationships between molecular characteristics and clinical outcomes.

## Methods

### Patients

A total of 413 newly diagnosed MM patients, diagnosed from 1995 to 2016, were included in the present study. Most of them, 319 (77%), were enrolled in clinical trials from the GEM-PETHEMA Spanish MM group: GEM2000^19^ (NCT00560053, *n* = 27), GEM2005más65^20^ (NCT00443235, *n* = 23), GEM2005MENOS65^21^ (NCT00461747, *n* = 20), GEM2010MAS65^22^ (NCT01237249, *n* = 83), QUIREDEX^23^ (NCT00480363, *n* = 29), GEM2012MENOS65^24^ (NCT01916252, *n* = 130) and GEM-CESAR^25^ (NCT00480363, *n* = 7). The 94 remaining patients were treated following the same protocols (GEM2000, GEM2005), but they were not formally included in any clinical trial.

According to the International Myeloma Working Group (IMWG) criteria^26^, patients with either t(4;14), t(14;16) or del17p were grouped together as a high-risk (HR) cytogenetics subset for subsequent analysis. Patients with symptomatic myeloma were also stratified based on the Revised International Staging System (R-ISS)^27^.

This study was approved by the Ethical Committee of the University Hospital of Salamanca in accordance with the Spanish law and the Declaration of Helsinki principles. Written informed consent was obtained from every patient prior to the inclusion in each trial.

### Sample collection and IGH sequencing

Genomic DNA was isolated from bone marrow aspirates at the time of diagnosis using the automated DNA Purification kit Maxwell® (Promega, Madison, WI, USA). DNA quantification and quality assessment were done using the NanoDrop2000 Spectrophotometer (ThermoFisher, Waltham, MA, USA). For the amplification of complete *VDJH* rearrangements, we used the BIOMED-2 (now Euroclonality©) FR1 primers^28^ in multiplexed PCR reactions. All reactions were carried out in a 25 μL mixture containing ~100 ng DNA and 10 μmol of each primer. Monoclonal assessment of amplified products was carried out by GeneScanning using 1 μL of PCR reaction. PCR products were sequenced in an automated ABI3500 XL DNA sequencer using Big-Dye terminators v3.1 (Applied Biosystems™, Foster City, CA, USA).

One hundred and thirteen samples were also analyzed by next-generation sequencing (NGS) using the LymphoTrack® methodology (Invivoscribe Technologies, San Diego, CA, USA). We targeted the Framework Region 1 to amplify VDJH rearrangements from 113 patients. In one-step PCR, amplicons were generated and indexed. A purification step using Agentcourt AMPure XP microbeads (Beckman Coulter Inc, Brea, CA, USA) was performed; then, we assessed the quality of our amplicons using the TapeStation 4200 (Agilent, Santa Clara, CA, USA) and Qubit 2.0 (ThermoFisher, Waltham, MA, USA). Libraries were sequenced in an MiSeq platform (Illumina, San Diego, CA, USA) with 2 × 251 of read length and aiming for 1 million reads per sample.

### Immunophenotypic characterization

Patients were routinely characterized at diagnosis by multiparametric flow cytometry. Bone marrow samples were processed within 48 h after collection. Analysis was performed using different assays, following the EuroClonality/EuroFlow consortium guidelines: six-color panel for six markers (CD38/CD138/CD45/CD19/CD56/CD117) in 78 patients, eight-color panel for eight markers (CD38/CD138/CD45/CD19/CD56/CD117/CD27/CD81) in the remaining 335 patients.

For data analysis, the values of all parameters measured per tube were mathematically calculated for the individual plasma-cell events using the merge and calculation functions of the INFINICYT™ v2.0 software (Cytognos S.L. Salamanca, Spain). Plasma cells were identified based on the characteristic pattern of expression of CD38, CD138, CD45 and light scatter features.

### Sequence analysis

*IGHV*, *IGHD* and *IGHJ* genes from complete rearrangements detected using either Sanger or NGS were identified comparing them with the IMGT/V-Quest database^29^, taking into account the option for insertions/deletions. Mutational status was assessed using the closest germline gene. Sequences containing more than 2% deviation from the germline were considered somatically mutated, following previously described criteria^13^. Point mutations were annotated, as well as the length and composition of N-, P- and CDR3 regions.

FastQ files generated by deep sequencing were processed using the LymphoTrackAnalysis® tool to retrieve sequences from virtually every clonal B cell in the samples, allowing the identification of tumor clones if the following criteria were met: (i) ≥20,000 total reads; (ii) at least one but not more than 2 merged top reads ≥ 2.5% of total reads and (iii) top first or second merged reads must be at least twice more abundant than the third most frequent read to be considered clonotypic.

Rearrangements were considered biallelic if the first two reads met the aforementioned criteria, had similar read count (less than 50% difference) and one was productive while the other one unproductive. Moreover, rearrangements were considered potentially biclonal if the first two reads met the aforementioned criteria, had similar read count (less than 50% difference) and both were productive.

### CDR3 clustering analysis

ClustalX2.0 was used as previously described^30,31^, comparing CDR3 amino acid sequences obtained from our cohort and 1117 additional sequences obtained from the IMGT/LIGM-DB database^32^ corresponding to unique rearrangements from both normal and human tumor B cells.

Primary and secondary standard criteria^31,33^ were used as described before to characterize immunoglobulin clusters.

### Statistical analysis

Clinical and biological characteristics were analyzed discriminating between transplant-eligible and noneligible patients, as well as asymptomatic patients, with the SPSS 20.0 software (IBM, Armonk, NY, USA) using Fisher’s exact test for discrete variables and the Mann−Whitney test for continuous variables. The Kaplan–Meier method and the log-rank test were used to plot and compare progression-free survival (PFS) and overall survival (OS) curves. Cox regression was used to perform the multivariate analysis. Receiver operating characteristic (ROC) curves were used to evaluate potential cutoff values for some variables. PFS was defined as the time from diagnosis to either disease progression, death or the last follow-up visit. OS was defined as the time from diagnosis to the last follow-up visit or decease. All reported *p* values were obtained by a two-sided exact method, at the conventional 5% significance level (*p* < 0.05).

## Results

### Clinical characteristics

Baseline characteristics of the 413 patients included in the study are summarized in Table 1.

**Table 1:** Patient characteristics.

Variable	Global series (*N* = 413)	Transplant-eligible (*N* = 228)	Transplant-ineligible (*N* = 149)	Asymptomatic (*N* = 36)
Sex				
Men	54.5%	62.1%	45.8%	54.8%
Women	45.5%	37.9%	54.2%	45.2%
Age	64 years (37−91)	59 years (37−69)	72 years (48−91)	60 years (42−77)
IgH				
IgG	59.4%	55.8%	62.8%	42.3%
IgA	32.8%	35.5%	30.4%	50%
Bence−Jones	6.9%	7.0%	6.8%	7.7%
Nonsecretory	0.9%	1.7%	0%	0%
IgL				
Kappa chain	62.5%	63.5%	60.7%	50%
Lambda chain	37.5%	36.5%	39.3%	50%
Calcium	9.63 ± 1.72 mg/dL (0.45−17)	9.50 ± 1.99 mg/dL (0.45−17)	9.78 ± 1.37 mg/dL (2.28−14.4)	9.11 ± 0.49 mg/dL (8−10)
Creatinine	1.16 ± 0.84 mg/dL (0.20−6.50)	1.11 ± 0.76 mg/dL (0.20−6.50)	1.21 ± 0.93 mg/dL (0.36−5.90)	0.89 ± 0.28 mg/dL (0.50−1.61)
Albumin	3.52 ± 0.67 g/dL (1.43−5.50)	3.54 ± 0.73 g/dL (1.70−5.14)	3.51 ± 0.61 g/dL (1.43−5.50)	3.76 ± 0.46 g/dL (2.20−4.60)
β2 microglobulin	5.17 ± 5.22 mg/L (0−62)	5.11 ± 5.45 mg/L (0−62)	5.20 ± 4.98 mg/L (0.15−43.40)	2.25 ± 1.13 mg/L (0.30−4.41)
Hemoglobin	10.54 ± 1.98 g/dL (4.90−15.60)	10.55 ± 1.99 g/dL (5.50−15.50)	10.56 ± 1.97 g/dL (4.90−15.60)	12.38 ± 1.13 g/dL (10.10−14.70)
R-ISS^a^				
Stage I	19.1%	24.1%	13.1%	61.9%
Stage II	69.7%	62.8%	77.9%	38.1%
Stage III	11.2%	13.1%	9.0%	0%
ECOG				
0	23.8%	27.6%	20.3%	66.7%
1	49.7%	49.3%	50.7%	33.3%
2	20.2%	17.8%	21.6%	0%
3	4.0%	4.6%	3.4%	0%
4	2.3%	0.7%	4.1%	0%
Bone lesions				
None	19.2%	19.7%	18.9%	100%
Minor lesions	42.1%	45.4%	38.5%	0%
Major lesions	38.7%	34.9%	42.7%	0%
Plasmacytoma	11.2%	14.1%	8.3%	0%
High LDH	15%	16.6%	13.3%	5.4%
t(11;14)	13.2%	7.4%	18.1%	3.4%
t(4;14)	11.7%	12.2%	9.4%	19.4%
t(14;16)	3%	4.6%	1%	6.9%
17p abnormalities	7%	7.1%	7.2%	4.3%
HR cytogenetics	20.3%	21.6%	16.5%	29.6%
1q gain	47.7%	46.4%	50%	26.3%
del1p	5.7%	7.2%	3%	20%

Clinical features were compared distinguishing between symptomatic (transplant-candidates and noncandidates) and smoldering myeloma patients.

*IgH* immunoglobulin heavy chain, *IgL* immunoglobulin light chain, *R-ISS* Revised International Staging System, *ECOG* Eastern Cooperative Oncology Group Performance Status, *LDH* lactate dehydrogenase, *HR cytogenetics* high-risk cytogenetics.

^a^Proportions of each ISS group are shown instead of the R-ISS for asymptomatic myeloma patients.

For transplant-eligible patients (*n* = 228), R-ISS stages I, II and III represented 24.1%, 62.8% and 13.1% of this subset, respectively. After induction, before the pretransplant conditioning regimen started, the overall response ratio (ORR) was 92.4%: 15.3% were in stringent complete response (sCR); 24.1% of patients had achieved complete response (CR); and 26.5% of patients were in very good partial response (VGPR). The remaining patients were in partial response (PR, 26.5%). Median PFS was 49.5 months; median OS was 67.9 months.

For transplant-ineligible patients (*n* = 149), R-ISS stages I, II and III represented 13.1%, 77.9% and 9% of this subgroup, respectively. After induction, the ORR was 89.7%: 7.7% were in sCR, 34.2% achieved CR, 14.5% were in VGPR and 33.3% were in PR. Median PFS was 26.4 months; median OS was 57.7 months.

As expected, high-risk asymptomatic patients (*n* = 36) were younger (median age: 60 years). Serum albumin levels were higher (median: 3.76 g/dL) and β2 microglobulin levels were lower (median: 2.25 mg/L). Consequently, 61.9% of smoldering patients were at ISS stage I, and 38.1% were at stage II.

### Detection of clonal VDJH gene rearrangements

Sanger sequencing allowed the identification of full clonal *VDJH* gene rearrangements in 88% of patients (363/413). In 23 cases, the clonal *IGH* gene rearrangement was not detected, possibly due to a high polyclonal background or lack of PCR amplification due to an incorrect primer-annealing. In 12 cases, we could only identify the *IGHV* gene, while in the remaining cases we could identify two out of three genes (mostly *IGHV* and *IGHJ*, but not *IGHD*, *n* = 13).

**Fig. 1:**
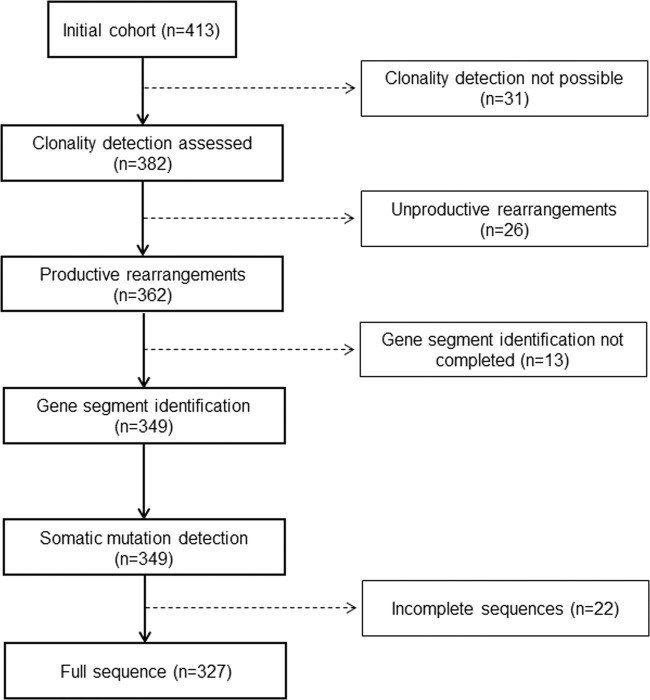
Study flowchart. From the starting population (*n* = 413), 92 patients were excluded. Hence, molecular profiling and survival analyses were performed with 327/413 patients (79.2%).

Combining Sanger and NGS, the overall *VDJH* detection rate was 92.5% (382/413). In all, 388 complete rearrangements were detected, including the identification of five cases with a biallelic rearrangement, as well as one case with a potential biclonal myeloma that was later confirmed by flow cytometry (Fig. S1). Among them, 362 rearrangements (93.3%) were productive, while 26 rearrangements (6.7%) were unproductive (Fig. 1). Six of these unproductive sequences corresponded to light-chain myeloma patients.

### Gene repertoire

*IGHV*, *IGHD* and *IGHJ* usage was identified in productive rearrangements. Table 2 shows the frequencies of *IGHV* gene group usage among the 362 productive rearrangements, with observed and expected frequencies (based on the number of genes per *IGHV* group), as well as SHM rates. No major differences were observed comparing transplant and nontransplant subgroups.

**Table 2:** Group usage and SHM rates in IGHV genes.

IGHV group	Expected frequency	Observed frequency	Median SHM % (95% CI)
*IGHV1*	10 (18.2%)	55 (15.2%)	10.0 (8.6–10.5)
*IGHV2*	3 (5.4%)	31 (8.6%)	6.5 (6.0–8.0)
*IGHV3*	26 (47.3%)	191 (52.7%)	8.8 (8.7–9.7)
*IGHV4*	12 (21.8%)	67 (18.5%)	9.2 (9.2–11.4)
*IGHV5*	2 (3.6%)	18 (5.0%)	7.2 (6.1–9.0)
*IGHV6*	1 (1.8%)	0	–
*IGHV7*	1 (1.8%)	0	–
Total	55	362	8.8 (8.8–9.6)

Comparison of SHM %	Adjusted *p* value		
*IGHV1* vs*. IGHV2*	0.006		
*IGHV2* vs. *IGHV4*	0.001		
*IGHV2* vs. *IGHV3*	0.012		
*IGHV1* vs. *IGHV5*	0.301		
*IGHV4* vs*. IGHV5*	0.118		
*IGHV3* vs. *IGHV5*	0.790		
*IGHV1* vs. *IGHV4*	1		
*IGHV2* vs. *IGHV5*	1		
*IGHV3* vs. *IGHV4*	1		
*IGHV1* vs. *IGHV3*	1		

The first two columns show the expected IGHV gene usage if all genes were randomly selected compared with the observed frequency within our cohort, respectively. Observed and expected IGHV distributions were not different when the *χ*^2^ test was applied, demonstrating that there is no evidence of selection of specific gene groups in myeloma. Median somatic hypermutation rates per *IGHV* group are shown on the right side of the table. Below, Kruskal−Wallis-based paired comparisons are listed to determine potential differences in the hypermutation rate; a significantly lower mutation rate was found for patients using *IGHV2* compared to those using *IGHV1* (*p* = 0.006), *IGHV3* (*p* = 0.012) and *IGHV4 (p* = 0.001).

*95% CI* 95% confidence intervals.

#### IGHV genes

According to the IMGT criteria^29,32^, we detected 50 functional genes (Fig. S2 and Table S1) in our cohort. Five canonical *IGHV* genes were never seen: *IGHV1-45, IGHV3-20, IGHV4-28, IGHV6-1* and *IGHV7-04*. Overall, *IGHV3-30* was the most abundant *IGHV* gene, with 46/362 cases (12.7%), followed by *IGHV3-33* (5.2%), *IGHV3-23* (5%) and *IGHV1-69* (4.7%). On the other hand, some *IGHV* genes usually rearranged in the normal B-cell repertoire, such as *IGHV3-20* or *IGHV3-74*, were underrepresented or totally absent. Another interesting finding was the presence of four cases using the *IGHV4-34* gene: two of them had high SHM rates (10 and 18.9%), both were unproductive, and patients had light-chain myeloma; the other two cases also showed higher SHM rates than the mean (20.8 and 16.7%) and clonal rearrangements were productive. One of these patients had light-chain myeloma while the other one was IgG secretory MM.

Finally, we also found noncanonical, haplotype-dependent *IGHV* genes in ten patients: *IGHV4-30-2* (*n* = 1), *IGHV4-30-4* (*n* = 5), *IGHV4-38-2* (*n* = 1) and *IGHV5-10-1* (*n* = 3), and one pseudogene (*IGHV3-69-1*, *n* = 1).

Compared to previous myeloma series*, IGHV* gene frequencies in our cohort were similar with three exceptions (*IGHV3-20*, *IGHV4-30-2* and *IGHV6-1)* that were not found in our patients. We also observed that, while *IGHV3* gene group representation was similar between both series, *IGHV3-30* was overrepresented and *IGHV3-30-3* was underrepresented in our cohort.

#### IGHD and IGHJ genes

*IGHD* and *IGHJ* gene identification was assessed in 349/362 sequences (96%).

*IGHD3* and *IGHD2* groups were the most abundant (30.4% and 25.4%, respectively), with *IGHD3-10* (10%) and *IGHD2-21* (8%) genes significantly overrepresented (*p* < 0.05) as compared to the expected value in healthy plasma cells. Conversely, *IGHD4-4* and *IGHD6-25* were not found in any sequence, and all members from the *IGHD1* gene group but *IGHD1-26* seemed to be underrepresented (less than 1.5% each one, Fig. S3 and Table S2). All these features were similar in previous myeloma series and were also seen in other hematologic B-cell malignancies and in normal plasma cells, although preference for *IGHD2-21* was specially marked only in myeloma.

For *IGHJ* gene usage, we found a deep bias in the use of certain genes matching previous observations concerning different hematologic malignancies such as CLL, MCL, and the normal bone marrow compartment: a significant overrepresentation of *IGHJ4* (46.4%) and *IGHJ6* (25%) was estimated (Fig. S4 and Table S3), while *IGHJ2* and *IGHJ1* were clearly underrepresented (2.9% and 1.1%, respectively). However, we noticed that, compared to previous series in myeloma, *IGHJ4* was underrepresented and *IGHJ6* overrepresented. In addition, *IGHJ1* and *IGHJ6* usage in healthy plasma cells looked completely different not only with respect to myeloma, but also with other mature B-cell disorders.

We explored associations between *VDJH* repertoire and risk factors. *IGHJ6* gene group was more frequent among patients with high-risk cytogenetics (37.9% vs. 17.8%, *p* = 0.025) considering only transplant-candidate patients. *IGHD1* gene group was also more frequently used by patients harboring high-risk cytogenetics, although this association was restricted to patients not candidates for transplantation (41.7% vs. 13.2%, *p* = 0.026). However, there were not relevant associations between the usage of these genes and the outcome in terms of PFS or OS.

Transplant-eligible and -ineligible patients showed similar proportions in *VDJH* gene usage (Table S4). In the same line, high-risk smoldering patients did not show significant differences in the use of *VDJH* genes compared to symptomatic patients.

### Somatic hypermutation rates and *IGH* composition

Somatic mutations could be correctly identified in 349 sequences: mutational load was high (mean: 9.2% ± 3.8; median: 8.8%; 95% CI: 8.8−9.6%; range: 0−22.7%), with five cases showing more than 98% of homology with germline genes. Comparing SHM rates between *IGHV* groups, we found a significantly lower rate for *IGHV2* compared to *IGHV1, IGHV3* and *IGHV4* (Table 2). Median SHM ratios of transplant-eligible and -ineligible patients were similar (9.0% vs. 8.5% respectively, *p* = 0.869). Median SHM rate in symptomatic and asymptomatic patients was also similar (9.0% vs. 8.8%, *p* = 0.845). The complete sequence was available in 327/362 cases, so we continued the analysis with this set (Fig. 1).

As far as the junction region was concerned, nucleotide addition appeared in 92.5% of cases for the N1 region, and in 88.6% of cases for the N2 region. Only three cases did not show evidence of nucleotide addition, either in N1 or in N2. Mean Guanine/Cytosine content of the N-regions was 58.8%. Evidence of exonuclease activity in at least one of the two junction regions (*IGHV*-to-*IGHD* and/or *IGHD*-to-*IGHJ*) was observed in all rearrangements, although 32.2% of cases displayed a fully conserved *IGHV*-3′ edge.

#### CDR3 amino acid composition

According to the IMGT numbering, we identified CDR3 positions (from amino acid 105 to amino acid 117). Median CDR3 length was 15 amino acids (range 6−29); this length was consistent across different myeloma subgroups (symptomatic, asymptomatic, transplant-eligible and transplant-ineligible patients). Amino acid proportions in myeloma were not significantly different from the normal B-cell population (Fig. S5), with predominance of Glycine, Alanine, Aspartic acid and Tyrosine.

### Clustering analysis

CDR3 sequences from productive rearrangements (*n* = 362) were subjected to cluster analysis. We found one pair from our cohort that showed more than 60% of amino acid similarity. Consequently, and given they were clusters not previously described, we applied the secondary criteria. Although CDR3 lengths were similar (13 vs. 15 amino acids) and both rearrangements displayed the same IGHJ4*02 germinal gene, *IGHV* and *IGHD* groups, as well as *IGHD* reading frames, were not concordant. Thus, we could not find any stereotyped immunoglobulin cluster among these myeloma patients.

### Impact of molecular variables on clinical outcomes

Considering patients with symptomatic myeloma, after a median follow-up of 7.6 years (range 0.5−24.1), 120 patients had died; the majority of them (*n* = 76) were transplant-ineligible. ROC curves showed that 7 and 8% were the most optimal cutoffs for prognostic value of SHM; optimal cutoffs for serum albumin (3.2 g/dL), β2 microglobulin (3.5 mg/dL), hemoglobin (9 g/dL) and creatinine (1 mg/dL) were also estimated. In addition, *IGHD2* and *IGHD3* users were grouped for subsequent analyses, because they were the most common *IGHD* groups and this bias has been observed in nearly every B-cell malignancy^9–18^. Finally, all molecular data described in previous paragraphs were combined with clinical data to perform statistical comparisons in transplant-eligible and -ineligible patients.

We first looked for relationships between treatment response and molecular variables. No major associations were seen, but two exceptions: (1) SHM ≥ 7% associated with a higher rate of CR/sCR compared to patients with SHM < 7% in the nontransplant subset (45.8% vs. 27.3%, respectively; *p* = 0.026), and (2) *IGHD3-3* usage was associated with a better response in the transplant subgroup (*n* = 10), since 80% of MM patients with an *IGH* rearrangement choosing this particular gene achieved CR/sCR vs. 36.8% for other *IGHD* genes (*p* = 0.019).

In addition, we detected that SHM rates were progressively higher when symptomatic patients were analyzed based on the differentiation stage of their pathologic plasma cells: median SHM levels for CD19+/CD81+ (immature), CD19−/CD81+ (intermediate) and CD19−/CD81− (mature) plasma-cell subsets were 7.0% (95% CI: 5.2−11.4%), 8.50% (95% CI: 7.9−9.6%), and 9.3% (95% CI: 9.1−10.2%), respectively (*p* = 0.029). Moreover, median PFS increased from intermediate to mature subgroups (35.4 vs. 64.0 months respectively, *p* = 0.024) but not from immature to intermediate subsets, probably because we only found 11 CD19+/CD81+ cases in our series.

#### Transplant-ineligible patients

**Fig. 2:**
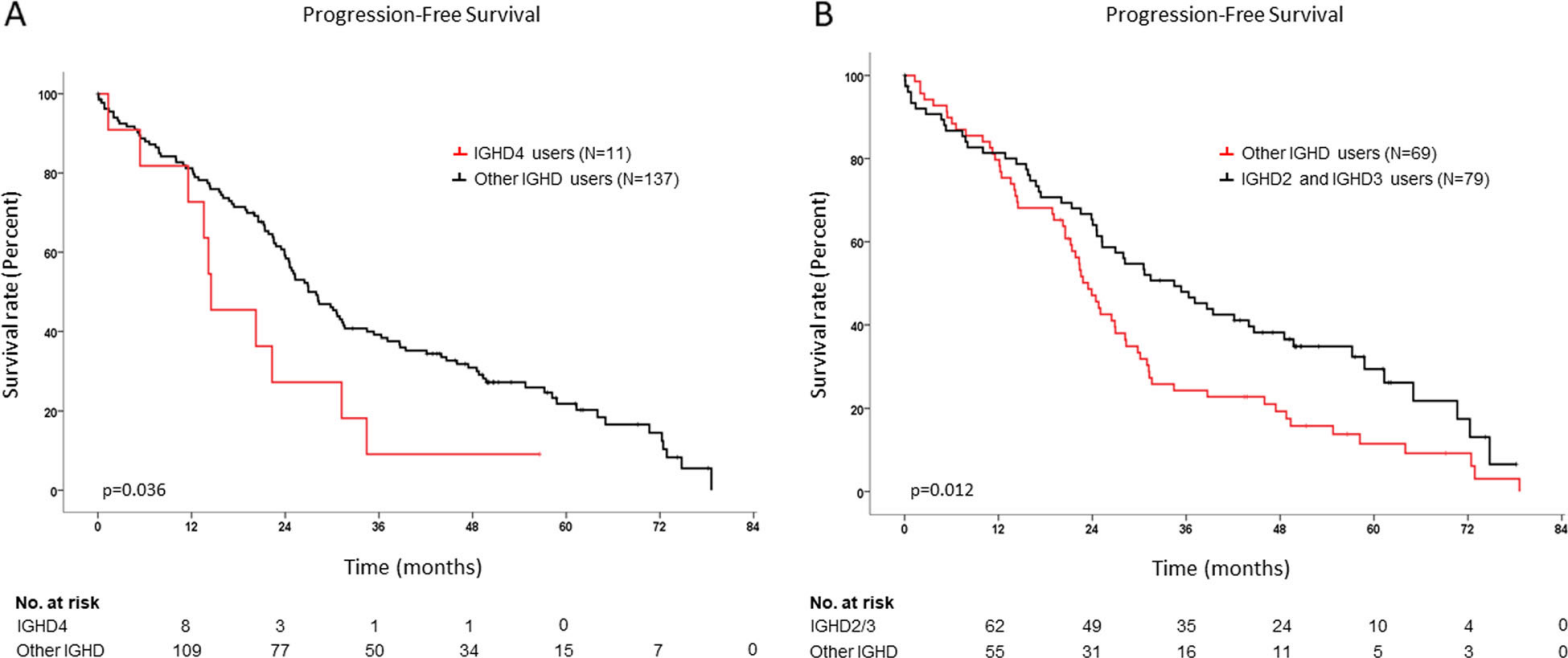
Kaplan−Meier curves comparing PFS for *IGHD4* and *IGHD2/3* users in transplant-ineligible patients. Survival curves compare progression-free survival rates between *IGHD4* (**a**) or *IGHD2/IGHD3* users (**b**) and other *IGHD* users. *IGHD4* users are represented in red, while *IGHD2/IGHD3* users are represented in black in their respective plots. Patients at risk at each time point appear below each plot.

**Fig. 3:**
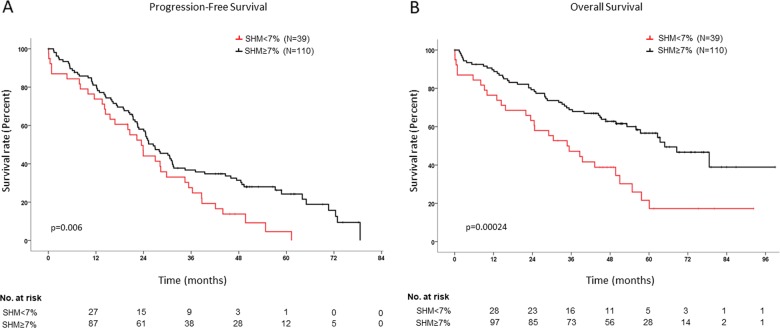
Kaplan−Meier curves comparing PFS and OS for SHM subsets in transplant-ineligible patients. **a** Progression-free survival plot. **b** Overall survival plot. Cases with more than 7% of SHM are represented in black, while cases with SHM below 7% are represented in red. Patients at risk at each time point appear below each plot.

In the univariate analysis for PFS, improved survival of transplant-ineligible patients was associated with 0−1 ECOG stages (*p* = 0.003), CR/sCR after the end of induction (*p* = 2 × 10^−6^), baseline hemoglobin ≥ 9 g/dL (*p* = 0.002), no use of the *IGHD4* gene group (*p* = 0.036) (Fig. 2a) or specifically the *IGHD4-11* gene (*p* = 0.0003), use of the *IGHD2/IGHD3* gene groups (*p* = 0.012) (Fig. 2b), and SHM rate ≥ 7% (*p* = 0.006) (Fig. 3a). Improved OS rates were observed in the univariate analysis for those patients with CR/sCR after induction (*p* = 0.00026), SHM rate ≥ 7% (*p* = 0.00024) (Fig. 3b), standard-risk cytogenetics (*p* = 0.031) and serum hemoglobin ≥ 9 g/dL (*p* = 0.002).

One hundred and eleven transplant-ineligible patients were included in the multivariate analysis. For PFS (Table 3), CR/sCR achievement (*p* = 10^−6^, HR: 0.311, 95% CI: 0.196−0.494) and the use of *IGHD2/IGHD3* gene groups (*p* = 0.006, HR: 0.552, 95% CI: 0.361−0.845) appeared as independent prognostic factors of prolonged survival rates. For OS, CR achievement after induction retained its independent prognostic value for improved survival probability (*p* = 0.010, HR: 0.347, 95% CI: 0.154−0.780) as well as SHM ≥ 7% (*p* = 0.001, HR: 0.291, 95% CI: 0.137−0.618) and standard cytogenetic risk (*p* = 0.016, HR: 0.369, 95% CI: 0.164−0.832). Moreover, SHM rate was included in the multivariate regression model not only as a discrete variable but also as a continuous one to avoid potential bias induced with the cutoff selection of 7%; in this case, the variable retained its prognostic value (*p* = 0.017, HR: 0.909, 95% CI: 0.840−0.983) along with achieving CR/sCR and the absence of cytogenetic risk, showing that an increment of 1% in SHM reduces death risk by 9%.

**Table 3:** Univariate and multivariate analysis of clinical and biological factors influencing PFS and OS of transplant-ineligible patients in our cohort.

Variable	Univariate analysis for PFS		Multivariate analysis for PFS		Univariate analysis for OS		Multivariate analysis for OS	
	Median survival	*p*	HR [95% CI]	*p*	Median survival	*p*	HR [95% CI]	*p*
ECOG score								
0−1	26.94	0.003		NS		NS		
2−4	21.35							
Serum hemoglobin								
<9 g/dL	14.03	0.001		NS	24.54	0.002		NS
≥9 g/dL	28.32				64.85			
Post induction response								
sCR/CR	49.35	0.008	0.311 [0.196−0.494]	1E−05	Not reached	0.00026	0.347 [0.154−0.780]	0.010
VGPR/PR	23.88				53.19			
Cytogenetic risk								
High risk	36.30	0.196		NS	78.59	0.031	0.369 [0.164−0.832]	0.016
Standard risk	25.03				39.95			
IGHD4 usage								
Yes	14.49	0.036		NS		NS		
No	27.96							
IGHD4-11 usage								
Yes	14.16	0.0003		NS		NS		
No	26.94							
IGHD2 and IGHD3 usage								
Yes	34.46	0.012	0.552 [0.361−0.845]	0.006		NS		
No	23.46							
SHM level								
<7%	23.46	0.006		NS	34.73	0.00024	0.291 [0.137−0.618]	0.001
≥7%	26.45				64.85			

Univariate analysis was first performed to evaluate the role of each clinical and biological variable described in this study. The table only shows statistically significant variables that were later included in the multivariate analysis. Median survival is shown in months.

*HR* hazard ratio, *CI* confidence interval, *NS* nonsignificant.

#### Transplant-eligible patients

Univariate analysis for PFS showed improved outcomes for patients in R-ISS stage I vs. II (*p* = 0.002) and III (*p* = 0.004), standard-risk cytogenetics (*p* = 3 × 10^−6^), 0−1 ECOG stages (*p* = 0.015), absence of 1q gains (*p* = 0.019) and serum β2 microglobulin < 3.5 mg/dL (*p* = 0.023). The R-ISS stage (*p* = 6 × 10^−6^), high-risk cytogenetics (*p* = 4 × 10^-6^), the ECOG score (*p* = 0.004), 1q gains (*p* = 0.016) and serum β2 microglobulin (*p* = 0.002) also showed significant associations with the OS in the same direction as PFS. Interestingly, no molecular variable was significantly associated with survival in this subset of patients.

One hundred and thirty-seven transplant-eligible patients were included in the multivariate analysis. Presence of 1q gains (*p* = 0.046, HR: 2.187, 95% CI: 1.015−4.714) and high-risk cytogenetics (*p* = 0.000021, HR: 4.690, 95% CI: 2.267−9.700) were associated with lower PFS rates. High-risk cytogenetics retained its independent prognostic value for shorter OS (*p* = 0.001, HR: 8.030, 95% CI: 2.406−26.809), while serum β2 microglobulin levels < 3.5 mg/dL associated with better outcomes (*p* = 0.039, HR: 0.116, 95% CI: 0.015−0.899).

## Discussion

Here, we analyzed in depth the *IGH* locus, trying to correlate its characteristics with clinical data in a large cohort of 413 MM patients included in clinical trials. *VDJH* usage description provided new insights into the clonal differentiation and possible clinical implications for MM, that could potentially improve patient care.

*IGHV* gene repertoire reflected its normal counterpart, with *IGHV3* being the predominant gene group. As in other series, *IGHV3* was overrepresented and *IGHV1* underrepresented, although differences were not significant, with a much less pronounced bias than that observed in CLL^12,13,31^. Similarly, *IGHV3-30, IGHV4-59* and *IGHV4-39* were overrepresented with respect to other *IGHV* genes^34–36^, and although these genes are also common in other lymphoproliferative disorders and normal plasma cells, we noted that *IGHV3-30* was specifically selected only in myeloma. In contrast, *IGHV3-23* and *IGHV1-69* appeared to be less frequently selected in MM (≥8% of representation in the normal B-cell population^35,36^ and B-cell pathologies vs. ≤5% in our series).

*IGHV4-34* representation is high in hematologic malignancies such as B-ALL, CLL, MCL or diffuse large B-cell lymphoma as well as in normal B cells^9,11,31^. Interestingly, this gene is usually absent in the normal plasma-cell population^37^, although it shows a wide interindividual usage frequency^38^, and it is rarely selected in MM^17^, but in this work it was not completely absent. *IGHV4-34* has been characterized as an inherently autoreactive antibody^39,40^ for it is widely represented in autoimmune diseases (i.e. systemic lupus erythematosus). Here, we found four clonal *IGHV4-34* rearrangements; they showed very high SHM rates and were usually nonproductive, leading to light-chain myelomas. This could be explained by the fact that during plasma-cell differentiation, those cells displaying autoreactive-prone genes within their IGH rearrangements would be specially targeted by the somatic hypermutation machinery, trying to make them able to recognize only foreign antigens. Moreover, in our series, two out of the three unproductive rearrangements using *VH1-69*, another gene commonly associated with autoimmune events, such as Thrombotic Thrombocytopenic Purpura^41^, and highly represented in CLL, were light-chain myelomas with some of the highest SHM rates in our cohort (mean 12.23%). Interestingly, this gene is commonly unmutated in CLL patients^42,43^.

SHM level was high and similar to what has been observed in other studies for healthy and pathological plasma and memory B cells. This would be in line with the post-germinal center origin of myeloma^8^. A striking finding was the presence of five MM cases with no apparent SHM; however, the *IGHV* region could not be completely sequenced (the entire FWR1 and part of the FWR2 was missing); from our experience, we think the mutational load was most likely underestimated, rather than being truly unmutated cases.

CDR3 composition resembled the normal immunoglobulin repertoire, with similar length, amino acid use, or evidence of TdT and exonuclease activity^44^. This composition diverges from CLL, MCL or MZL patterns, where restricted antibody sets account for approximately 30% of cases^45–47^. Accordingly, cluster analysis failed to demonstrate the presence of stereotyped receptors in our cohort, as it has been shown before in myeloma patients^48^. Overall, the gene repertoire in our series was very close to previous reports^17,18,48^, although functional *IGHV* and *IGHD* genes, whose use has not been reported in myeloma to date, have been found in this cohort probably due to its larger size. On the contrary, other genes such as *IGHV1-18*, *IGHV3-20*, *IGHV4-30-2* and *IGHV6-1*, found in Italian^48^ or Greek^18^ patients, are absent in Spanish patients, maybe reflecting differences in the normal repertoire between different Mediterranean subpopulations. No differences were observed in the median length of the CDR3 region, and somatic hypermutation was slightly higher than in previous publications; however, this variation was not statistically significant.

The most remarkable finding was the association between a higher SHM level and an improved survival rate. The explanation for this situation seems to be connected with the maturation stage of tumor cells, represented by the expression of CD19 and CD81 biomarkers, which is consistent with previous findings regarding the immune profile of tumor plasma cells:^49^ it suggests that early genetic alterations in the ontogeny of germinal-center B cells would provide a more aggressive profile in MM, which would be strong enough to overcome SHM and/or CSR earlier. Conversely, the acquisition of late, more harmless genetic events would allow tumor B cells to undergo SHM and CSR processes and this would lead to better outcomes. Another possible explanation is related to the proliferation ratio of tumor cells: highly proliferative B cells (and therefore, more aggressive clones) activate DNA repair pathways that are able to reduce the SHM level, as it has been shown in CLL patients^50,51^. However, the SHM cutoff is based on FR1 primers; this threshold could differ when using leader primers.

Another striking feature was the correlation between the use of *IGHD2* and *IGHD3* gene groups and the outcome. An increased usage of these particular groups has also been observed in other series suggesting a positive selective pressure over them^8,17,18^, since the bias was observed only for complete rearrangements. Nevertheless, whether or not the relationship between *IGHD* usage and survival is a myeloma characteristic remains uncertain.

SHM and *IGHD* usage were associated with the outcome only in the elderly, transplant-ineligible patients. Thereby, the effect of intensive regimens could be the main reason to explain why the negative prognostic impact of these molecular characteristics is diluted in younger, fit patients.

Regarding the value of clinical variables on the patient outcome, the prognostic role of cytogenetics, hemoglobin, albumin, β2 microglobulin and ECOG in MM was confirmed^52^. Cytogenetic risk has to be mentioned as this variable showed significant associations with survival in all cases but when applied to PFS in transplant-ineligible patients. The absence of FISH studies for most patients diagnosed from 1995 to 2000 (most of them being older) made possible to find only 17 patients with high-risk cytogenetics in this subgroup, which could explain this result.

In conclusion, we have shown that multiple myeloma plasma cells resemble the normal mature B-cell repertoire but, in contrast to CLL, MCL or MZL, stereotyped receptors were absent^14,15,53,54^. Light-chain and nonsecretory myeloma cases could be explained by incorrect *IGH* rearrangements at the genomic DNA level, but not always, which means that other effects could hamper the immunoglobulin production, including transcriptional or translational defects. Finally, here, and for the first time, we have reported how some molecular characteristics of the IGH gene rearrangement, such as higher SHM rates or *IGHD2/IGHD3* gene usage, could positively impact on the outcome of patients. A validation series is currently planned to elucidate whether these findings could be applicable in real-life patients; if validated in independent studies, they could be considered as new molecular markers for PFS and OS in multiple myeloma.

## Supplementary information

Supplemental material

## References

[1] Early P, Huang H, Davis M, Calame K, Hood L (1980). An immunoglobulin heavy chain variable region gene is generated from three segments of DNA: VH, D and JH. Cell.

[2] Oettinger MA, Schatz DG, Gorka C, Baltimore D (1990). RAG-1 and RAG-2, adjacent genes that synergistically activate V(D)J recombination. Science.

[3] Komori T, Okada A, Stewart V, Alt FW (1993). Lack of N regions in antigen receptor variable region genes of TdT-deficient lymphocytes. Science.

[4] Matsuda F (1998). The complete nucleotide sequence of the human immunoglobulin heavy chain variable region locus. J. Exp. Med..

[5] Tonegawa S (1983). Somatic generation of antibody diversity. Nature.

[6] Mostoslavsky R, Alt FW, Rajewsky K (2004). The lingering enigma of the allelic exclusion mechanism. Cell.

[7] Honjo T, Kinoshita K, Muramatsu M (2002). Molecular mechanism of class switch recombination: linkage with somatic hypermutation. Annu. Rev. Immunol..

[8] González D (2007). Immunoglobulin gene rearrangements and the pathogenesis of multiple myeloma. Blood.

[9] Hockley SL (2012). The prognostic impact of clinical and molecular features in hairy cell leukaemia variant and splenic marginal zone lymphoma. Br. J. Haematol..

[10] Petrikkos L (2014). Clonotypic analysis of immunoglobulin heavy chain sequences in patients with Waldenström’s macroglobulinemia: correlation with MYD88 L265P somatic mutation status, clinical features, and outcome. BioMed. Res. Int..

[11] Mroczek ES (2014). Differences in the composition of the human antibody repertoire by B cell subsets in the blood. Front. Immunol..

[12] Marinelli M (2016). Immunoglobulin gene rearrangements in Chinese and Italian patients with chronic lymphocytic leukemia. Oncotarget.

[13] Hamblin TJ, Davis Z, Gardiner A, Oscier DG, Stevenson FK (1999). Unmutated Ig V(H) genes are associated with a more aggressive form of chronic lymphocytic leukemia. Blood.

[14] Agathangelidis A (2012). Stereotyped B-cell receptors in one-third of chronic lymphocytic leukemia: a molecular classification with implications for targeted therapies. Blood.

[15] Darzentas N, Stamatopoulos K (2013). Stereotyped B cell receptors in B cell leukemias and lymphomas. Methods Mol. Biol..

[16] Kiyoi H, Naito K, Ohno R, Naoe T (1996). Comparable gene structure of the immunoglobulin heavy chain variable region between multiple myeloma and normal bone marrow lymphocytes. Leukemia.

[17] González D (2005). Molecular characteristics and gene segment usage in IGH gene rearrangements in multiple myeloma. Haematologica.

[18] Hadzidimitriou A (2006). Immunoglobulin genes in multiple myeloma: expressed and non-expressed repertoires, heavy and light chain pairings and somatic mutation patterns in a series of 101 cases. Haematologica.

[19] Lahuerta JJ (2010). Busulfan 12 mg/kg plus melphalan 140 mg/m2 versus melphalan 200 mg/m2 as conditioning regimens for autologous transplantation in newly diagnosed multiple myeloma patients included in the PETHEMA/GEM2000 study. Haematologica.

[20] Mateos MV (2010). Bortezomib, melphalan, and prednisone versus bortezomib, thalidomide, and prednisone as induction therapy followed by maintenance treatment with bortezomib and thalidomide versus bortezomib and prednisone in elderly patients with untreated multiple myeloma: a randomised trial. Lancet Oncol..

[21] Rosinol L (2012). Superiority of bortezomib, thalidomide, and dexamethasone (VTD) as induction pretransplantation therapy in multiple myeloma: a randomized phase 3 PETHEMA/GEM study. Blood.

[22] Mateos MV (2016). Sequential vs alternating administration of VMP and Rd in elderly patients with newly diagnosed MM. Blood.

[23] Mateos MV (2016). Lenalidomide plus dexamethasone versus observation in patients with high-risk smouldering multiple myeloma (QuiRedex): long-term follow-up of a randomised, controlled, phase 3 trial. Lancet Oncol..

[24] Rosinol L (2019). Bortezomib, lenalidomide, and dexamethasone as induction therapy prior to autologous transplant in multiple myeloma. Blood.

[25] Mateos MV (2013). Lenalidomide plus dexamethasone for high-risk smoldering multiple myeloma. N. Engl. J. Med..

[26] Chng WJ (2014). IMWG consensus on risk stratification in multiple myeloma. Leukemia.

[27] Palumbo A (2015). Revised International Staging System for multiple myeloma: a report from International Myeloma Working Group. J. Clin. Oncol..

[28] van Dongen JJM (2003). Design and standardization of PCR primers and protocols for detection of clonal immunoglobulin and T-cell receptor gene recombinations in suspect lymphoproliferations: report of the BIOMED-2 Concerted Action BMH4CT98-3936. Leukemia.

[29] Brochet X, Lefranc M-P, Giudicelli V (2008). IMGT/V-QUEST: the highly customized and integrated system for IG and TR standardized V-J and V-D-J sequence analysis. Nucleic Acids Res..

[30] Larkin MA (2007). Clustal W and Clustal X version 2.0. Bioinformatics..

[31] Stamatopoulos K (2007). Over 20% of patients with chronic lymphocytic leukemia carry stereotyped receptors: pathogenetic implications and clinical correlations. Blood..

[32] Lefranc MP (2009). IMGT, the international ImMunoGeneTics information system. Nucleic Acids Res..

[33] Messmer BT (2004). Multiple distinct sets of stereotyped antigen receptors indicate a role for antigen in promoting chronic lymphocytic leukemia. J. Exp. Med..

[34] Kosmas C (2000). Origin and diversification of the clonogenic cell in multiple myeloma: lessons from the immunoglobulin repertoire. Leukemia.

[35] Brezinschek HP (1997). Analysis of the human VH gene repertoire. Differential effects of selection and somatic hypermutation on human peripheral CD5(+)/IgM+ and CD5(-)/IgM+ B cells. J. Clin. Invest..

[36] Kraj P (1997). The human heavy chain Ig V region gene repertoire is biased at all stages of B cell ontogeny, including early pre-B cells. J. Immunol..

[37] Pugh-Bernard AE (2001). Regulation of inherently autoreactive VH434 B cells in the maintenance of human B cell tolerance. J. Clin. Invest..

[38] Turchaninova M A, Davydov A, Britanova O V, Shugay M, Bikos V, Egorov E S, Kirgizova V I, Merzlyak E M, Staroverov D B, Bolotin D A, Mamedov I Z, Izraelson M, Logacheva M D, Kladova O, Plevova K, Pospisilova S, Chudakov D M (2016). High-quality full-length immunoglobulin profiling with unique molecular barcoding. Nature Protocols.

[39] Bhat NM, Lee LM, van Vollenhoven RF, Teng NNH, Bieber MM (2002). VH4-34 encoded antibody in systemic lupus erythematosus: effect of isotype. J. Rheumatol..

[40] Mockridge CI (2004). Common patterns of B cell perturbation and expanded V4-34 immunoglobulin gene usage in autoimmunity and infection. Autoimmunity.

[41] Pos W (2009). VH1-69 germline encoded antibodies directed towards ADAMTS13 in patients with acquired thrombotic thrombocytopenic purpura. J. Thromb. Haemost..

[42] Duke VM (2003). V(H) gene usage differs in germline and mutated Bcell chronic lymphocytic leukemia. Haematologica..

[43] González-Gascón Y (2014). Mutation status and immunoglobulin gene rearrangements in patients from northwest and central region of Spain with chronic lymphocytic leukemia. BioMed. Res. Int..

[44] Shi B (2014). Comparative analysis of human and mouse immunoglobulin variable heavy regions from IMGT/LIGM-DB with IMGT/HighV-QUEST. Theor. Biol. Med. Model..

[45] Darzentas N (2010). A different ontogenesis for chronic lymphocytic leukemia cases carrying stereotyped antigen receptors: molecular and computational evidence. Leukemia.

[46] Agathangelidis A, Hadzidimitriou A, Rosenquist R, Stamatopoulos K (2011). Unlocking the secrets of immunoglobulin receptors in mantle cell lymphoma: implications for the origin and selection of the malignant cells. Semin. Cancer Biol..

[47] Hadzidimitriou A (2011). Is there a role for antigen selection in mantle cell lymphoma? Immunogenetic support from a series of 807 cases. Blood.

[48] Ferrero S (2012). Multiple myeloma shows no intra-disease clustering of immunoglobulin heavy chain genes. Haematologica.

[49] Paiva B (2017). Differentiation stage of mieloma plasma cells: biological and clinical significance. Leukemia.

[50] van Gent R (2008). In vivo dynamics of stable chronic lymphocytic leukemia inversely correlate with somatic hypermutation levels and suggest no major leukemic turnover in bone marrow. Cancer Res..

[51] Giné E (2010). Expanded and highly active proliferation centers identify a histological subtype of chronic lymphocytic leukemia (“accelerated” chronic lymphocytic leukemia) with aggressive clinical behavior. Haematologica.

[52] Rajkumar SV (2016). Multiple myeloma: 2016 update on diagnosis, risk-stratification, and management. Am. J. Hematol..

[53] Varettoni M (2013). Clues to pathogenesis of Waldenström macroglobulinemia and immunoglobulin M monoclonal gammopathy of undetermined significance provided by analysis of immunoglobulin heavy chain gene rearrangement and clustering of B-cell receptors. Leuk. Lymphoma.

[54] Zibellini S (2010). Stereotyped patterns of B-cell receptor in splenic marginal zone lymphoma. Haematologica.

