# Supplementary material for: Molecular profiling of immunoglobulin heavy-chain gene rearrangements unveils new potential prognostic markers for multiple myeloma patients
Source: Blood Cancer J. 2020 Feb 6;10(2):14. doi: 10.1038/s41408-020-0283-8 (PMC7004993; doi:10.1038/s41408-020-0283-8)
Supplement: Supplementary file 1 — Supplemental material [file 41408_2020_283_MOESM1_ESM.docx]

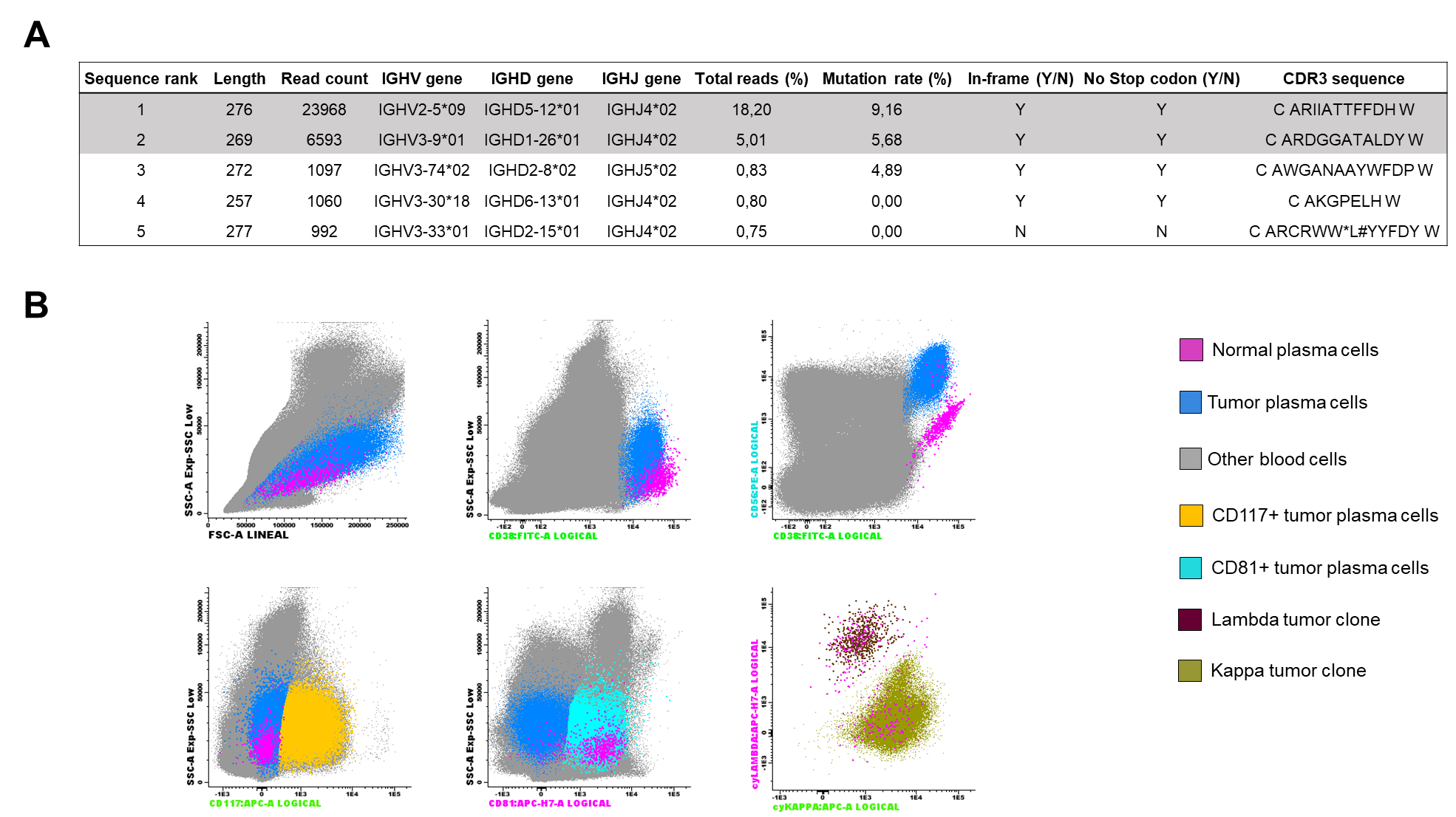
Supplemental Figure 1.

**Supplemental Figure 1. Biclonal Myeloma Confirmation by Next-Generation Sequencing and Multiparametric Flow Cytometry.** A) Next-generation sequencing results. The table shows top-5 clonotypes following FR1 sequencing. Two clonotypic rearrangements met criteria to be considered independent tumor clones: gene usage, rearrangement length, CDR3 and SHM rates were different; both were productive and represented at least 5% of total reads. B) The bone marrow sample was analyzed using the following 2-tube 8-color antibody panel: CD38, CD138, CD45, CD19, CD27, CD28, CD56, CD81, CD117, CyIgκ, CyIgλ and β2-microglobulin. Previously standardized EuroFlow methods were applied. Normal and tumor plasma cells (in pink and dark blue, respectively) were identified by CD38 and CD56 expression, and distinguished from other blood cells (in grey). Bimodal expression of CD117 (50% Negative/50% Positive, in yellow) and CD81 markers (82% Negative/18% Positive, in sky blue) revealed two distinct tumor plasma cell subsets. The main clone was Kappa, while a small secondary Lambda clone was also identified. Y: Yes; N: No.

Supplemental Figure 2.


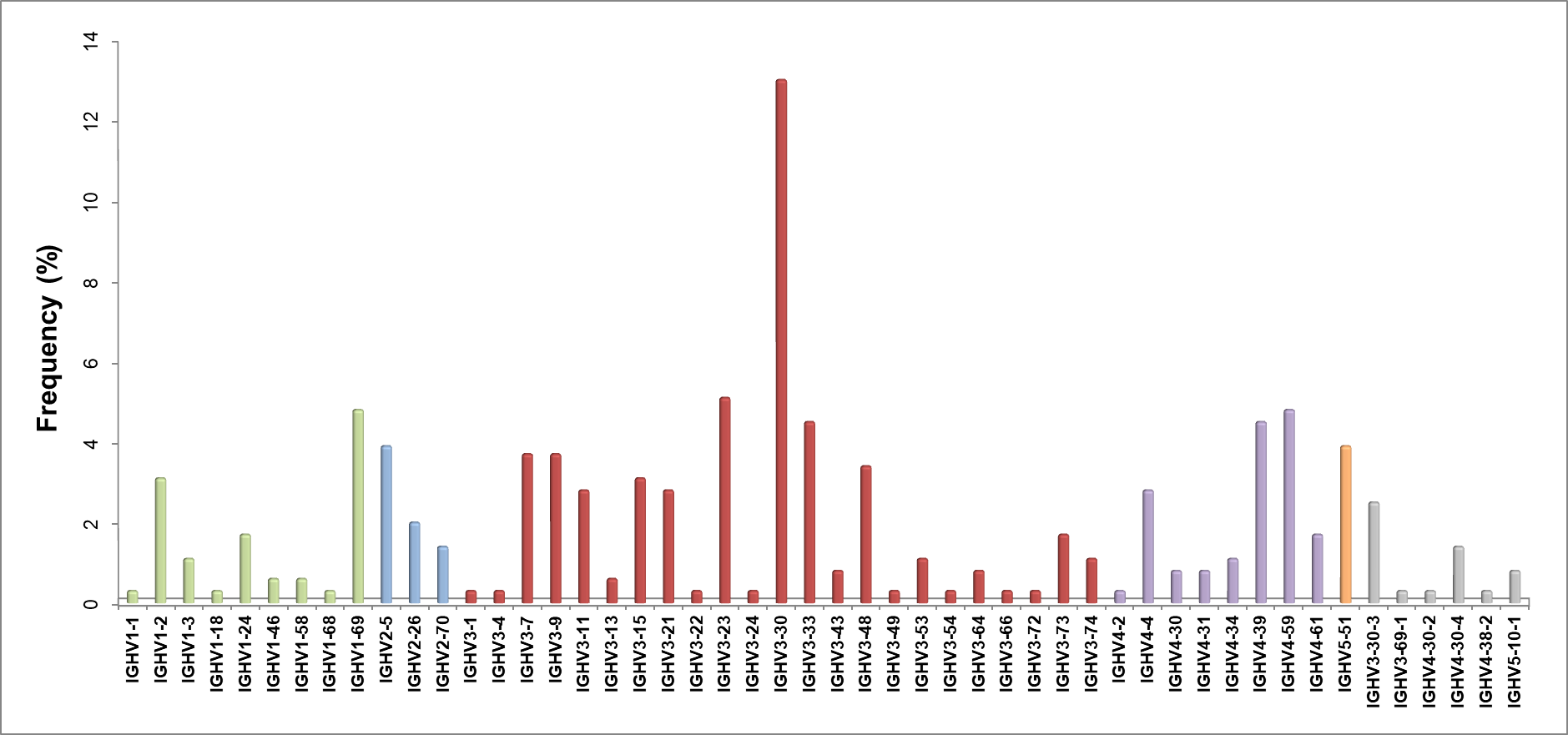


**Supplemental Figure 2. *IGHV* Gene Repertoire Analysis.** The barplot represents the 50 functional, canonical *IGHV* genes that were found in our cohort, as well as haplotype-dependent *IGHV* segments and pseudogenes (marked in grey).

Supplemental Figure 3.

**
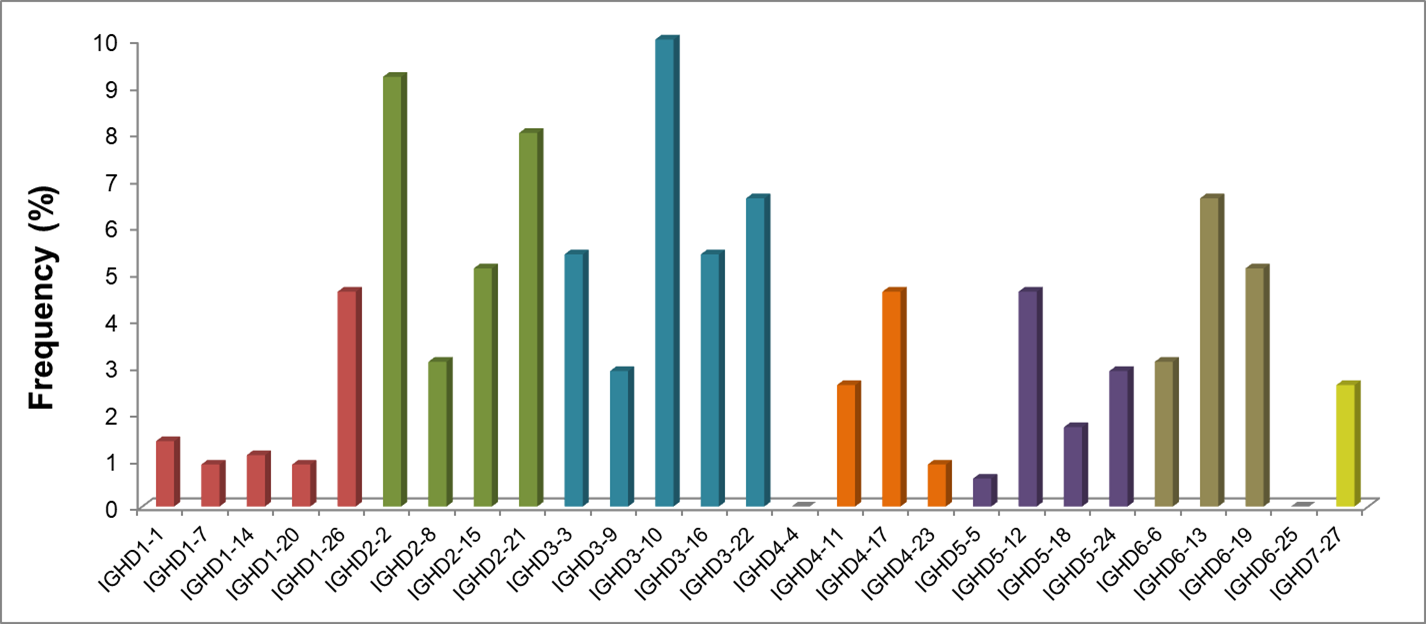
**

**Supplemental Figure 3. *IGHD* Gene Repertoire Analysis.** The barplot represents the 27 functional, canonical *IGHD* genes described in the literature. 25 of them (92.6%) were found in our cohort.

Supplemental Figure 4.

**
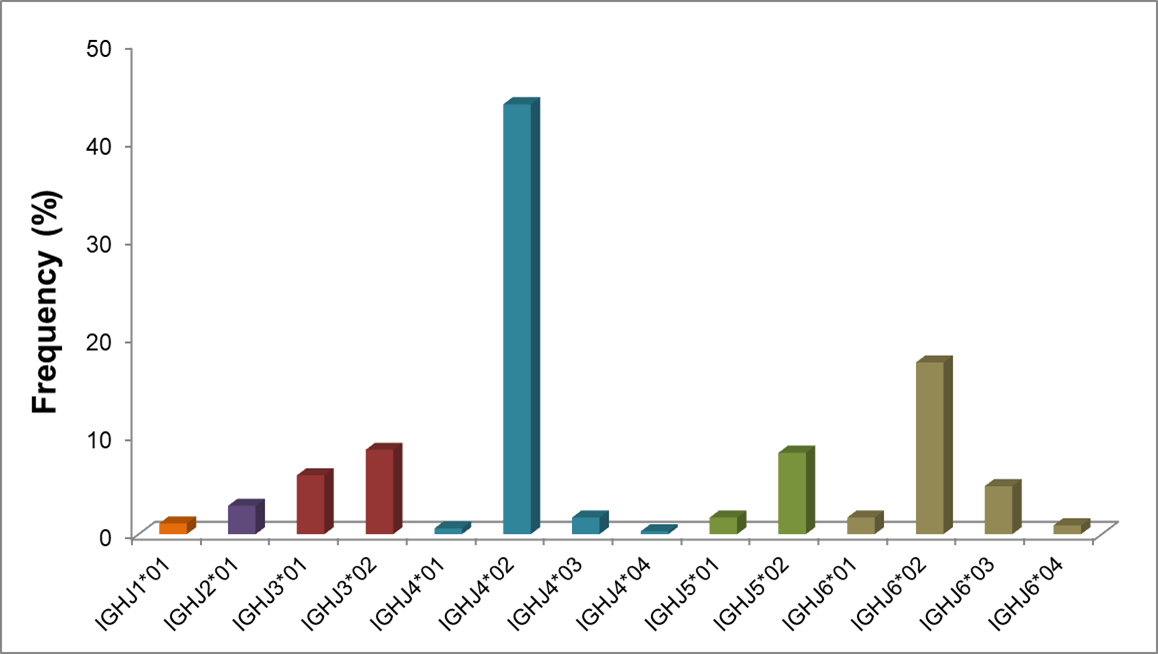
**

**Supplemental Figure 4. *IGHJ* Gene Repertoire Analysis.** The barplot represents the IGHJ genes reported in the literature. IGHJ4 and IGHJ6 gene groups were enriched in our series (46.4% and 25%, respectively), and mainly represented by *02 alleles (IGHJ4*02: 94.4% and IGHJ6*02: 70% of abundance in their respective groups).

**
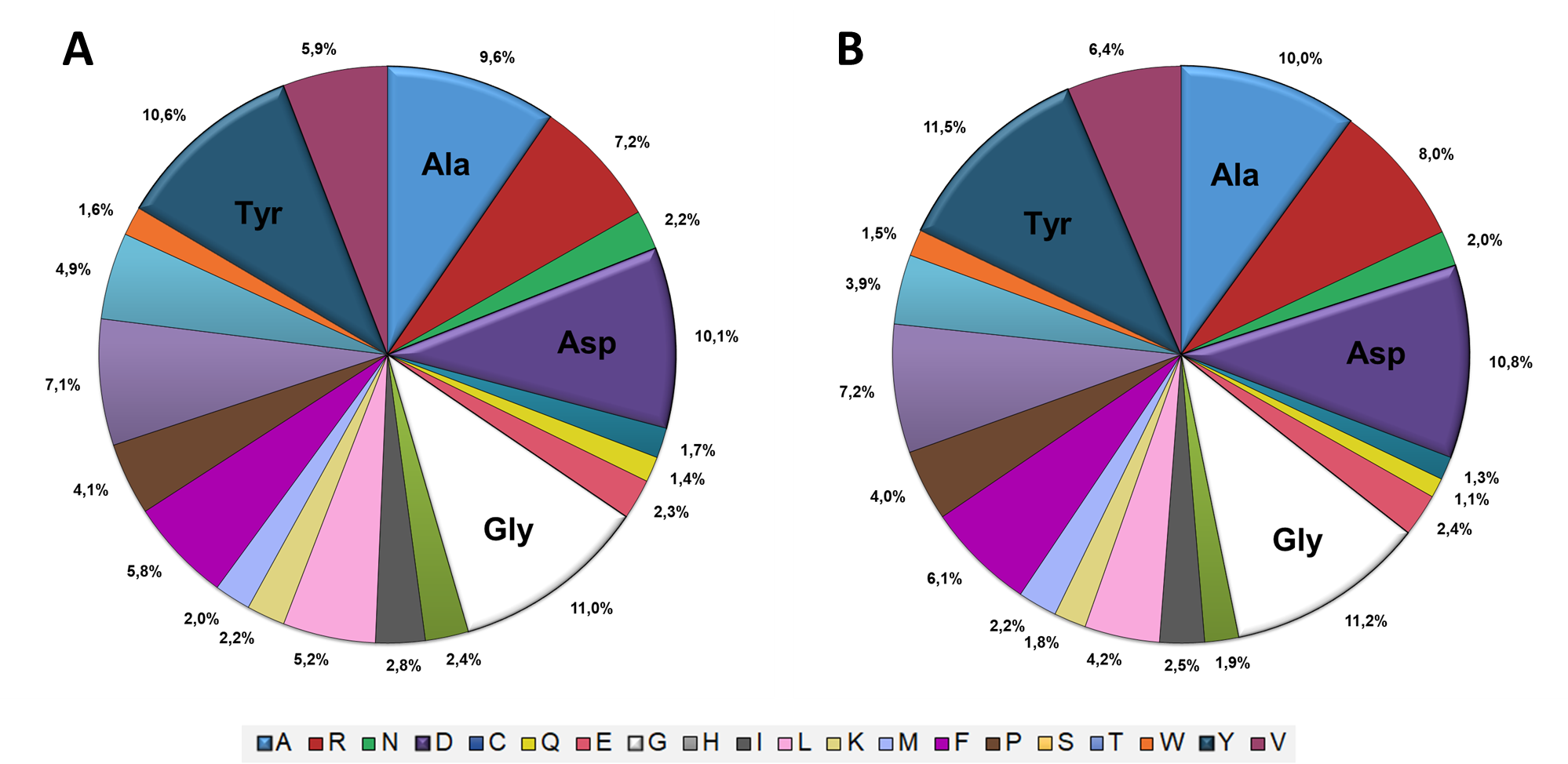
**Supplemental Figure 5.

**Supplemental Figure 5. CDR3 Amino Acid Composition in Myeloma and Normal B-Cells.** (A) Amino acid proportions in a “myeloma-arquetypal” CDR3 sequence based on the rearrangements of the 362 productive rearrangements in our cohort. (B) Frequencies of each amino acid in a subset of 9,340 normal B-cell CDR3 regions reported by Shi et al.^42^ The overall amino acid composition was similar between the two groups (χ2 test, 19 degrees of freedom, p>0.9), with a high correlation coefficient (R^2^: 0.987). No differences were observed comparing frequencies of individual amino acids between myeloma and normal B-cell rearrangements (χ2 test, 1 degree of freedom, p>0.05 for all paired comparisons).

| **IGHV allele** | **Medina et al. (N=362)** | **Other MM series (N=278)** | **CLL (N=7596)** | **MCL (N=807)** | **WM (N=58)** | **MZL (N=133)** | **CD19+/CD20-/CD38high plasma cells (N=1530)** |
| --- | --- | --- | --- | --- | --- | --- | --- |
| IGHV1-1 | 0.3% (1) | 0% (0) | 0% (0) | 0% (0) | 0% (0) | 0% (0) | 0% (0) |
| IGHV1-2 | 2.7% (10) | 1.8% (5) | 4.6% (348) | 2% (16) | 5.3% (3) | **19.5% (26)**** | 2.2% (34) |
| IGHV1-3 | 1.1% (4) | 1.1% (3) | 2.1% (158) | 1.7% (14) | 1.7% (1) | 0.7% (1) | 2.1% (32) |
| IGHV1-8 | 0.3% (1) | 0% (0) | 1% (77) | **7.8% (63)**** | 0% (0) | 0% (0) | 0% (0) |
| IGHV1-18 | 3.3% (12) | 1.4% (4) | **1.4% (109)*** | 2.1% (17) | 0% (0) | 2.2% (3) | 3.3% (51) |
| IGHV1-24 | 1.7% (6) | 1.8% (5) | **0.3% (26)**** | **0% (0)**** | 0% (0) | 0% (0) | **0.3% (4)**** |
| IGHV1-45 | 0% (0) | 0% (0) | 0.0% (2) | 0% (0) | 0% (0) | 0% (0) | 0% (0) |
| IGHV1-46 | 0.5% (2) | 0.4% (1) | 1.2% (96) | 0.4% (3) | 0% (0) | 0% (0) | 2% (30) |
| IGHV1-58 | 0.3% (1) | 0% (0) | 0.2% (18) | 0% (0) | 0% (0) | 0% (0) | 0% (0) |
| IGHV1-68 | 0.3% (1) | 0% (0) | 0% (0) | 0% (0) | 0% (0) | 0% (0) | 0% (0) |
| IGHV1-69 | 4.7% (17) | 5% (14) | **12.8% (973)**** | **1.5% (12)**** | 0% (0) | 7.5% (10) | 2.6% (40) |
| **IGHV1 total** | 15.2% (55) | 11.5% (32) | **23.8% (1807)**** | 15.5% (125) | 7.0% (4) | **30.1% (40)**** | 12.5% (191) |
| IGHV2-5 | 4.4% (16) | 2.5% (7) | 2.7% (205) | **0.9% (7)**** | 0% (0) | **0% (0)*** | **2.3% (35)*** |
| IGHV2-26 | 1.9% (7) | 2.1% (6) | **0.4% (24)**** | **0.4% (3)**** | 0% (0) | 0% (0) | **0.0% (1)**** |
| IGHV2-70 | 2.2% (8) | 2.5% (7) | **0.4% (26)**** | **0.3% (2)**** | 0% (0) | 0.7% (1) | **0.4% (6)**** |
| **IGHV2 total** | 8.5% (31) | 7.1% (20) | **3.4% (255)**** | **1.5% (12)**** | **0% (0)*** | **0.7% (1)**** | **2.7% (42)**** |
| IGHV3-1 | 0.3% (1) | 0% (0) | 0% (0) | 0% (0) | 1.7% (1) | 0% (0) | 0% (0) |
| IGHV3-4 | 0.3% (1) | 0% (0) | 0% (0) | 0% (0) | 0% (0) | 0% (0) | 0% (0) |
| IGHV3-7 | 3.9% (14) | 2.5% (7) | 5.7% (435) | 3.7% (30) | 8.8% (5) | 3.7% (5) | 4.3% (66) |
| IGHV3-9 | 4.1% (15) | 3.9% (11) | **1.8% (135)**** | 2.5% (20) | 1.7% (1) | 0.7% (1) | **0% (0)**** |
| IGHV3-11 | 3.6% (13) | 3.2% (9) | 2.5% (191) | 2.7% (22) | 0% (0) | 1.5% (2) | **0.5% (8)**** |
| IGHV3-13 | 0.5% (2) | 0.4% (1) | 0.3% (25) | 0.5% (4) | 0% (0) | 0% (0) | 1.3% (20) |
| IGHV3-15 | 2.5% (9) | 5% (14) | 2.5% (191) | 1.5% (12) | 3.5% (2) | 0% (0) | **5.4% (83)*** |
| IGHV3-20 | 0% (0) | 1.1% (3) | 0.4% (31) | 0.1% (1) | 0% (0) | 0% (0) | 0.2% (3) |
| IGHV3-21 | 2.7% (10) | 5.8% (16) | 4.7% (356) | 16.5% (133) | 5.3% (3) | 3% (4) | 3.1% (48) |
| IGHV3-22 | 0.3% (1) | 0% (0) | 0% (0) | 0% (0) | 0% (0) | 0% (0) | 0% (0) |
| IGHV3-23 | 5.0% (18) | 6.1% (17) | **8.5% (647)*** | 7.4% (60) | **29.8% (17)**** | **18% (24)**** | **22.9% (351)**** |
| IGHV3-24 | 0.3% (1) | 0% (0) | 0% (0) | 0% (0) | 0% (0) | 0% (0) | 0% (0) |
| IGHV3-30 | 12.7% (46) | **7.2% (20)*** | **5.5% (416)**** | **3.5% (28)**** | 8.8% (5) | **6% (8)*** | **3.1% (48)**** |
| IGHV3-33 | 5.2% (19) | 3.2% (9) | **2.9% (222)*** | **1.5% (12)**** | 1.7% (1) | 3.7% (5) | 3.4% (52) |
| IGHV3-43 | 0.8% (3) | 1.8% (5) | 0.3% (25) | 0.5% (4) | 0% (0) | 0% (0) | 0% (0) |
| IGHV3-48 | 2.7% (10) | 3.2% (9) | 3.9% (295) | 3.6% (29) | 0% (0) | 3.7% (5) | **6.1% (94)*** |
| IGHV3-49 | 0.3% (1) | 0.7% (2) | 1% (76) | 0.5% (4) | 0% (0) | 0.7% (1) | **3.1% (48)**** |
| IGHV3-53 | 1.1% (4) | 0% (0) | 1.5% (112) | 1% (8) | 1.7% (1) | 0% (0) | **3% (46)*** |

| **IGHV allele** | **Medina et al. (N=362)** | **Other MM series (N=278)** | **CLL (N=7596)** | **MCL (N=807)** | **WM (N=58)** | **MZL (N=133)** | **CD19+/CD20-/CD38high plasma cells (N=1530)** |
| --- | --- | --- | --- | --- | --- | --- | --- |
| IGHV3-54 | 0.3% (1) | 0% (0) | 0% (0) | 0% (0) | 0% (0) | 0% (0) | 0% (0) |
| IGHV3-64 | 0.3% (1) | 1.4% (4) | 0.6% (49) | 0.4% (3) | 0% (0) | 0% (0) | **1.9% (29)*** |
| IGHV3-66 | 0.3% (1) | 0.4% (1) | 0.7% (57) | 1.2% (10) | 0% (0) | 1.5% (2) | 0% (0) |
| IGHV3-72 | 0.3% (1) | 0.4% (1) | 1.1% (86) | 0.4% (3) | **5.3% (3)*** | 0.7% (1) | 0.8% (12) |
| IGHV3-73 | 1.4% (5) | 0.7% (2) | **0.4% (27)*** | **0% (0)**** | 3.5% (2) | 0.7% (1) | 2.1% (32) |
| IGHV3-74 | 1.1% (4) | 1.8% (5) | 2.3% (177) | **3.2% (26)*** | 5.3% (3) | **4.5% (6)*** | **4% (62)**** |
| IGHV3-30-3 | 2.5% (9) | **6.5% (18)*** | 1.4% (104) | 0.9% (7) | 0% (0) | 0.7% (1) | **0.1% (2)**** |
| IGHV3-69-1 | 0.3% (1) | 0% (0) | 0% (0) | 0% (0) | 0% (0) | 0% (0) | 0.1% (2) |
| **IGHV3 total** | 52.8% (191) | 55.3% (154) | 48.2% (3658) | 51.5% (416) | **77.2% (44)**** | 49.6% (66) | **65.7% (1006)**** |
| IGHV4-2 | 0.3% (1) | 0.4% (1) | 0.9% (68) | 0.5% (2) | 0% (0) | 0% (0) | 0% (0) |
| IGHV4-4 | 2.7% (10) | 3.2% (9) | 1.6% (124) | **0.7% (6)*** | 0% (0) | 1.5% (2) | **1.2% (18)*** |
| IGHV4-28 | 0% (0) | 0% (0) | 0% (0) | 0% (0) | 1.7% (1) | 0% (0) | 0% (0) |
| IGHV4-30 | 0.8% (3) | 0% (0) | **0% (0)**** | **0% (0)*** | 1.7% (1) | 0% (0) | **0% (0)*** |
| IGHV4-31 | 1.4% (5) | 1.1% (3) | 0.8% (59) | 0.6% (5) | 0% (0) | 0% (0) | 1.3% (20) |
| IGHV4-34 | 0.5% (2) | 1.8% (5) | **8.9% (673)**** | **14.6% (118)**** | 0% (0) | 0% (0) | **2.1% (32)*** |
| IGHV4-39 | 4.3% (16) | 3.9% (11) | 3.7% (283) | 4.6% (37) | 5.3% (3) | 1.5% (2) | **0% (0)**** |
| IGHV4-59 | 4.7% (17) | 4.3% (12) | **2.7% (209)*** | 4.6% (37) | 1.7% (1) | 3.7% (5) | 3.6% (55) |
| IGHV4-61 | 1.7% (6) | 1.8% (5) | 1.2% (90) | **0.4% (3)*** | 3.5% (2) | 2.2% (3) | 1.8% (28) |
| IGHV4-30-2 | 0.3% (1) | 1.1% (3) | 0.2% (13) | 0% (0) | 0% (0) | 0% (0) | 0% (0) |
| IGHV4-30-4 | 1.3% (5) | 0.7% (2) | 0.6% (48) | 0% (0) | 0% (0) | 0.7% (1) | **0% (0)**** |
| IGHV4-38-2 | 0.3% (1) | 0% (0) | 0% (0) | 0% (0) | 0% (0) | 0% (0) | **2.7% (42)**** |
| **IGHV4 total** | 18.5% (67) | 18.3% (51) | 20.3% (1567) | **25.8% (208)*** | 14% (8) | **9.8% (13)*** | **12.7% (195)*** |
| IGHV5-51 | 4.1% (15) | 5.4% (15) | **2% (149)*** | 4.6% (37) | 1.7% (1) | 1.5% (2) | 3.2% (49) |
| IGHV5-10-1 | 0.8% (3) | 0.4% (1) | 0.6% (43) | 0.5% (4) | 0% (0) | 0.7% (1) | 0.8% (13) |
| **IGHV5 total** | 5.0% (18) | 5.8% (16) | **2.5% (192)*** | 5.1% (41) | 1.7% (1) | 2.2% (3) | 4% (62) |
| IGHV6-1 | 0% (0) | **1.8% (5)*** | **1.2% (88)*** | 0.6% (5) | 1.7% (1) | 0% (0) | **1.2% (18)*** |
| IGHV7-4 | 0% (0) | 0% (0) | 0.4% (29) | 0% (0) | 0% (0) | 0% (0) | 1% (16) |

**Supplemental Table 1. *IGHV* gene usage.** The table lists the 55 *IGHV* genes that are defined in the literature for different mature B-cell malignacies, 50 of them were found in our series (91%). *IGHV* frequencies in our cohort were compared to those found in other mature B-cell malignancies, as well as in previous myeloma series and healthy plasma cells. References for comparisons are as follows: other myeloma series^18,19,48^; CLL^14^; MCL^47^; WM^53^; MZL^54^; CD19+/CD20-/CD38high plasma cells from one healthy subject^38^. Statistically significant differences are depicted in bold.

**P*<0.05

***P*<0.005

MM: multiple myeloma; CLL: chronic lymphocytic leukemia; MCL: mantle cell lymphoma; WM: Waldenström’s macroglobulinemia; MZL: marginal zone lymphoma.

| **IGHD allele** | **Medina et al. (N=349)** | **Other MM series (N=268)** | **CLL (N=7498)** | **MCL (N=802)** | **WM (N=53)** | **MZL (N=112)** | **CD19+/CD20-/CD38high plasma cells (N=1501)** |
| --- | --- | --- | --- | --- | --- | --- | --- |
| IGHD1-1 | 1.4% (5) | 2.5% (7) | 1.8% (137) | 2.1% (17) | 2% (1) | 0.9% (1) | 2.9% (44) |
| IGHD1-7 | 0.9% (3) | 1.1% (3) | 1.2% (88) | 1.4% (11) | 0% (0) | 1.7% (2) | 0.9% (13) |
| IGHD1-14 | 1.1% (4) | 0.7% (2) | 0.7% (55) | 0.2% (2) | 6% (3) | 0% (0) | 1.3% (19) |
| IGHD1-20 | 0.9% (3) | 0% (0) | 0.2% (13) | 0.2% (2) | 0% (0) | 0% (0) | 1.6% (24) |
| IGHD1-26 | 4.6 (16) | 4% (11) | 4.3% (325) | 6.6% (53) | 0% (0) | 2.6% (3) | 5.4% (81) |
| **IGHD1 total** | 8.9% (31) | 8.3% (23) | 8.2% (618) | 10.5% (85) | 7% (4) | 5.2% (6) | 12% (181) |
| IGHD2-2 | 9.2% (32) | 7.3% (20) | 9.0% (672) | 7.6% (61) | 11% (6) | 6% (7) | 9.9% (148) |
| IGHD2-8 | 3.1% (11) | 3.3% (9) | 1.8% (138) | 1.4% (11) | 2% (1) | 4.3% (5) | 4.3% (65) |
| IGHD2-15 | 5.1% (18) | 4% (11) | 5.4% (406) | 6.5% (52) | 6% (3) | 2.6% (3) | 6.5% (97) |
| IGHD2-21 | 8% (28) | 5.5% (15) | **3.4% (255)**** | **2% (16)**** | **0% (0)*** | 2.6% (3) | **5% (75)*** |
| **IGHD2 total** | 25.4% (89) | 20.1% (55) | **19.6% (1471)*** | **17.5% (140)**** | 19% (10) | **15.5% (18)*** | 25.6% (385) |
| IGHD3-3 | 5.4% (19) | 6.9% (19) | **14.5% (1089)**** | **10.1% (81)*** | 8% (4) | **20.7% (24)**** | 6.3% (95) |
| IGHD3-9 | 2.9% (10) | 2.5% (7) | 3.0% (229) | 5.6% (45) | 6% (3) | 4.3% (5) | 2.9% (43) |
| IGHD3-10 | 10% (35) | 11.3% (31) | 8.3% (623) | 9.2% (73) | 8% (4) | 5.2% (6) | **6.3% (94)*** |
| IGHD3-16 | 5.4% (19) | 5.5% (15) | 5.5% (413) | **2.7% (22)*** | 6% (3) | **0% (0)*** | 4.9% (74) |
| IGHD3-22 | 6.6% (23) | 7.3% (20) | 8.9% (668) | 6.6% (53) | 0% (0) | **12.9% (15)*** | 4.8% (72) |
| **IGHD3 total** | 30.4% (106) | 33.5% (92) | **40.3% (3022)*** | 34.2% (274) | 26% (14) | **43.1% (50)*** | 28.2% (424) |
| IGHD4-4 | 0% (0) | 1.1% (3) | 1.1% (83) | 1.6% (13) | 0% (0) | 0.9% (1) | 0.6% (9) |
| IGHD4-11 | 2.6% (9) | 0.4% (1) | **0% (0)*** | 0% (0) | 0% (0) | 0% (0) | 2.3% (34) |
| IGHD4-17 | 4.6% (16) | 6.2% (17) | 3.0% (226) | 4% (32) | 6% (3) | 2.6% (3) | 4% (60) |
| IGHD4-23 | 0.9% (3) | 2.5% (7) | 2.2% (167) | **2.9% (23)*** | 2% (1) | **4.3% (5)*** | 0.5% (8) |
| **IGHD4 total** | 8.1% (28) | 10.2% (28) | 6.3% (476) | 8.5% (68) | 9% (4) | 7.7% (9) | 7.4% (111) |
| IGHD5-5 | 0.6% (2) | 4% (11) | 3.3% (245) | 4.2% (34) | 8% (4) | 3.4% (4) | 0.7% (10) |
| IGHD5-12 | 4.6% (16) | 4.4% (12) | 3.0% (223) | 2.7% (22) | 4% (2) | 3.4% (4) | 3.5% (53) |
| IGHD5-18 | 1.7% (6) | 0% (0) | 0% (0) | 0% (0) | 0% (0) | 0% (0) | 1.5% (22) |
| IGHD5-24 | 2.9% (10) | 2.5% (7) | 2.6% (194) | 1.4% (11) | 0% (0) | 2.6% (3) | 2.3% (34) |
| **IGHD5 total** | 9.8% (34) | 10.9% (30) | 8.8% (662) | 8.3% (67) | 9% (6) | 9.5% (11) | 7.9% (119) |
| IGHD6-6 | 3.1% (11) | 2.5% (7) | 2.2% (167) | 5.1% (41) | 8% (4) | 4.3% (5) | 2.1% (32) |
| IGHD6-13 | 6.6% (23) | 3.3% (9) | 5.1% (385) | 7.0% (56) | 4% (2) | 4.3% (5) | **3.7% (56)*** |
| IGHD6-19 | 5.1% (18) | 6.5% (18) | **8.2% (612)*** | 7.7% (62) | **15% (8)*** | **6% (7)*** | 5.5% (83) |
| IGHD6-25 | 0% (0) | 0.4% (1) | 0.4% (31) | 0.5% (4) | 0% (0) | 0.9% (1) | 1.1% (17) |
| **IGHD6 total** | 14.8% (52) | 12.7% (35) | 15.9% (1195) | **20.3% (163)*** | **26% (14)*** | 15.5% (18) | 12.5% (188) |
| IGHD7-27 | 2.6% (9) | 1.8% (5) | **0.7% (54)**** | **0.6% (5)*** | 2% (1) | 0% (0) | **0.9% (13)*** |

**Supplemental Table 2. *IGHD* gene usage.** The table lists the 27 *IGHD* genes that are defined in the literature for different mature B-cell malignacies, 25 of them were found in our series (92.6%). *IGHD* frequencies in our cohort were compared to those found in other mature B-cell malignancies, as well as in previous myeloma series and healthy plasma cells. References for comparisons are as follows: other myeloma series^18,19,48^; CLL^14^; MCL^47^; WM^53^; MZL^54^; CD19+/CD20-/CD38high plasma cells from one healthy subject^38^. Statistically significant differences are depicted in bold. *IGHD4-4* and *IGHD4-11*, as well as *IGHD5-5* and *IGHD5-18,* are not well distinguished and they have been compared as a single gene.

**P*<0.05

***P*<0.005

MM: multiple myeloma; CLL: chronic lymphocytic leukemia; MCL: mantle cell lymphoma; WM: Waldenström’s macroglobulinemia; MZL: marginal zone lymphoma.

| IGHJ allele | Medina et al. (N=349) | Other MM series (N=278) | CLL (N=7596) | MCL (N=807) | WM (N=57) | MZL (N=133) | CD19+/CD20-/CD38high plasma cells (N=1526) |
| --- | --- | --- | --- | --- | --- | --- | --- |
| IGHJ1*01 | 1.1% (4) | 1.8% % (5) | 1.8% (137) | 0.6% (5) | 0% (0) | 1.5% (2) | **12.8% (196)**** |
| IGHJ2*01 | 2.9% (10) | 3.2% (9) | 2.2% (171) | 3.5% (28) | 2% (4) | 0% (0) | 1.7% (27) |
| IGHJ3*01 | 6%(21) | - | - | - | 2% (1) | - | - |
| IGHJ3*02 | 8.6% (30) | - | - | - | 5% (3) | - | - |
| IGHJ3 total | 14.6% (51) | 12.2% (34) | **9.9% (753)*** | **7.6% (61)**** | 7% (4) | 14.3% (19) | 13.4% (205) |
| IGHJ4*01 | 0.6% (2) | - | - | - | 0% (0) | - | - |
| IGHJ4*02 | 43.8% (153) | - | - | - | 62% (34) | - | - |
| IGHJ4*03 | 1.7% (6) | - | - | - | 2% (1) | - | - |
| IGHJ4*04 | 0.3% (1) | - | - | - | 0% (0) | - | - |
| IGHJ4 total | 46.4% (162) | **56.1% (156)*** | 43.3% (3289) | 43.7% (353) | **64% (35)*** | 37.6% (50) | 41.6% (635) |
| IGHJ5*01 | 1.7% (6) | - | - | - | 0% (0) | - | - |
| IGHJ5*02 | 8.3% (29) | - | - | - | 13% (7) | - | - |
| IGHJ5 total | 10% (35) | 12.6% (35) | 10.5% (796) | **15.0% (121)*** | 13% (7) | **19.5% (26)*** | **14.8% (226)*** |
| IGHJ6*01 | 1.7% (6) | - | - | - | 0% (0) | - | - |
| IGHJ6*02 | 17.5% (61) | - | - | - | 13% (7) | - | - |
| IGHJ6*03 | 4.9% (17) | - | - | - | 0% (0) | - | - |
| IGHJ6*04 | 0.9% (3) | - | - | - | 0% (0) | - | - |
| IGHJ6 total | 25% (87) | **14.0% (39)**** | **32.2% (2450)**** | 29.6% (239) | **13% (7)*** | 27.1% (36) | **15.5% (237)**** |

**Supplemental Table 3. *IGHJ* gene usage.** The table lists the 14 *IGHJ* genes that are defined in the literature, all of them present in our series. *IGHJ* frequencies in our cohort were compared to those found in other mature B-cell lymphoproliferative disorders, as well as in previous myeloma series and normal plasma cells. References for comparisons are as follows: other myeloma series^18,19,48^; CLL^14^; MCL^47^; WM^53^; MZL^54^; CD19+/CD20-/CD38high plasma cells from one healthy subject^38^. Statistically significant differences are depicted in bold.

**P*<0.05

***P*<0.005

MM: multiple myeloma; CLL: chronic lymphocytic leukemia; MCL: mantle cell lymphoma; WM: Waldenström’s macroglobulinemia; MZL: marginal zone lymphoma.

| IGHV | Symptomatic (N=330) | Asymptomatic (N=32) | Transplant-eligible (N=199) | Transplant-ineligible (N=131) |
| --- | --- | --- | --- | --- |
| IGHV1 | 15.6% (51) | 9.1% (3) | 15.2% (30) | 16.1% (21) |
| IGHV2 | 7.1% (23) | 15.2% (5) | 6% (12) | 8.7% (11) |
| IGHV3 | 54.1% (179) | 60.6% (19) | 55.4% (111) | 51.6% (68) |
| IGHV4 | 17.6% (58) | 12.1% (4) | 17.4% (34) | 18% (24) |
| IGHV5 | 5.7% (19) | 3% (1) | 6% (12) | 5.6% (7) |
| IGHV6 | 0% (0) | 0% (0) | 0% (0) | 0% (0) |
| IGHV7 | 0% (0) | 0% (0) | 0% (0) | 0% (0) |
| IGHD | **Symptomatic (N=318)** | **Asymptomatic (N=31)** | **Transplant-eligible (N=192)** | **Transplant-ineligible (N=126)** |
| IGHD1 | 9.7% (31) | 3.4% (1) | 9.7% (19) | 9.8% (12) |
| IGHD2 | 25.1% (80) | 37.9% (12) | 28% (54) | 20.3% (26) |
| IGHD3 | 31.1% (99) | 31.0% (10) | 30.9% (59) | 32% (40) |
| IGHD4 | 7.5% (24) | 3.4% (1) | 7.4% (14) | 7.8% (10) |
| IGHD5 | 9.3% (30) | 13.8% (4) | 10.3% (20) | 7.8% (10) |
| IGHD6 | 14.6% (46) | 10.3% (3) | 12% (23) | 18.3% (23) |
| IGHD7 | 2.5% (8) | 0% (0) | 1.7% (3) | 3.9% (5) |
| IGHJ | **Symptomatic (N=318)** | **Asymptomatic (N=31)** | **Transplant-eligible (N=192)** | **Transplant-ineligible (N=126)** |
| IGHJ1 | 0.9% (3) | 3.4% (1) | 0.6% (1) | 1.3% (2) |
| IGHJ2 | 3% (10) | 6.9% (2) | 4.5% (9) | 0.7% (1) |
| IGHJ3 | 14.6% (46) | 6.9% (2) | 13.1% (25) | 16.4% (21) |
| IGHJ4 | 48.3% (154) | 37.9% (12) | 48.3% (93) | 48.7% (61) |
| IGHJ5 | 11% (35) | 10.3% (3) | 13.6% (26) | 7.2% (9) |
| IGHJ6 | 22% (70) | 34.5% (11) | 19.9% (38) | 25.7% (32) |

**Supplemental Table 4. *VDJH* gene usage in different myeloma subgroups.** *IGHV*, *IGHD* and *IGHJ* frequencies were compared between symptomatic and asymptomatic patients, as well as between transplant-eligible and –ineligible patients. Two-sided Fisher’s exact test showed no differences in *VDJH* proportions between subgroups.
